# Supplementary material for: Water-organizing motif continuity is critical for potent ice nucleation protein activity
Source: Nat Commun. 2022 Aug 26;13:5019. doi: 10.1038/s41467-022-32469-9 (PMC9418140; doi:10.1038/s41467-022-32469-9)
Supplement: Supplementary file 1 — Supplementary Information [file 41467_2022_32469_MOESM1_ESM.pdf]

Supplementary Information to Water-organizing motif continuity is critical for potent ice nucleation protein activity

Jordan Forbes[1], Akalabya Bissoyi[2], Lukas Eickhoff[3], Naama Reicher[4], Thomas Hansen[1], Christopher G. Bon[1], Virginia K. Walker[5], Thomas Koop[3], Yinon Rudich[4], Ido Braslavsky[2], Peter L. Davies[1*]

[1]Department of Biomedical and Molecular Sciences, Queen's University, Kingston, ON Canada K7L 3N6

[2]The Robert H. Smith Faculty of Agriculture, Food and Environment, Institute of Biochemistry, Food Science, and Nutrition, The Hebrew University of Jerusalem, Rehovot 7610001, Israel

[3]Bielefeld University, Faculty of Chemistry, D-33615 Bielefeld, Germany

[4]Department of Earth and Planetary Sciences, The Weizmann Institute of Science, Rehovot 7610001, Israel

[5]Department of Biology, Queen's University, Kingston, ON Canada K7L 3N6

These authors contributed equally: Akalabya Bissoyi, Lukas Eickhoff, Naama Reicher.

[*]Corresponding author: Peter L. Davies, peter.davies@queensu.ca

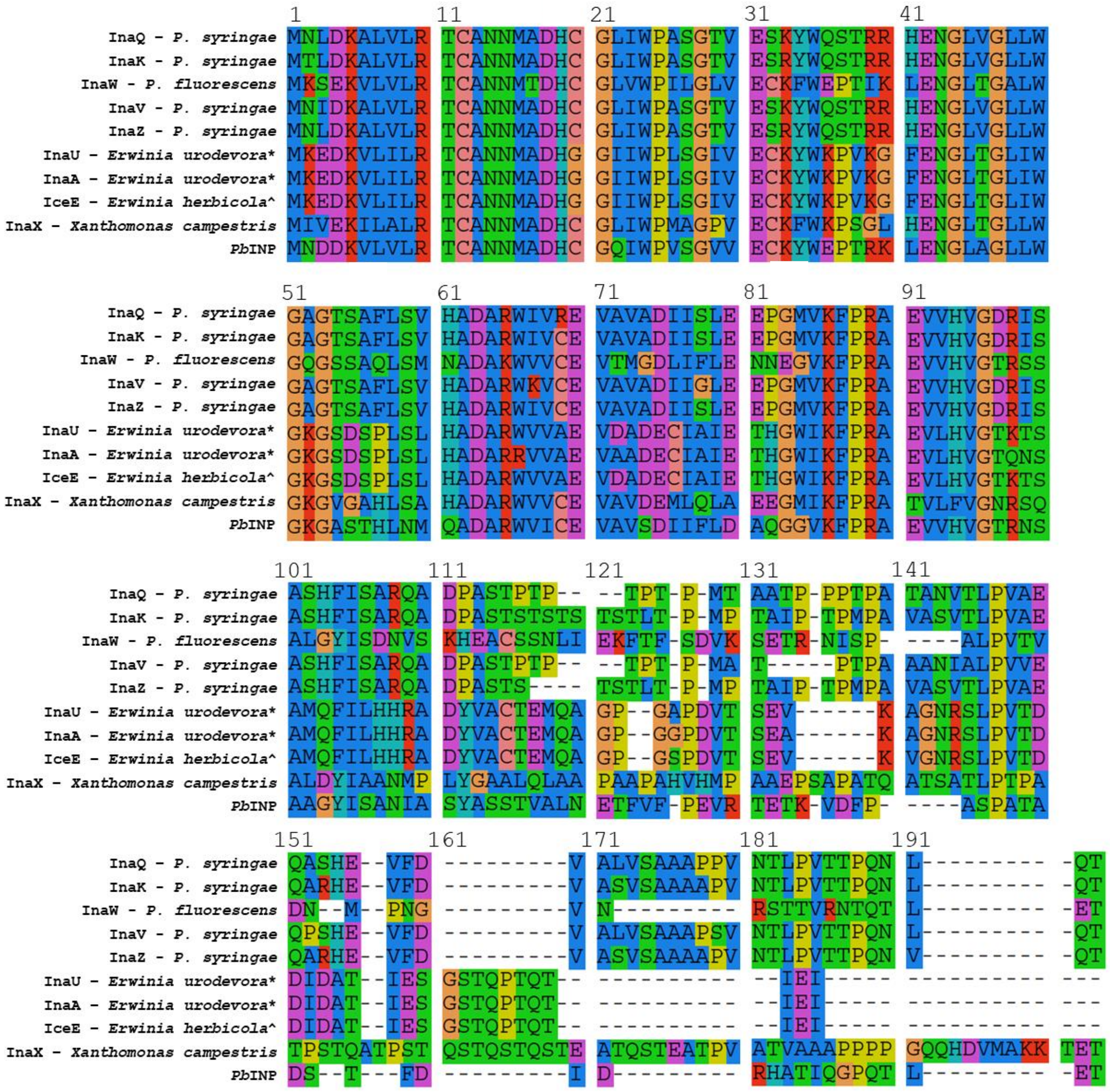

Supplementary Figure 1. INP N-terminal domain alignment. The annotated and reviewed INP genes are aligned, with gene and host species name on the left. Residues are identified by their one-letter code. Colouring is based on amino acid side chains: dark blue represents nonpolar, green is for polar uncharged aliphatic side chains, purple represents negatively charged, red is for positively charged, proline is yellow, histidine is light blue, and glycine is orange. **Erwinia urodevora* is sometimes referred to as *Pantoea ananas*. ^*Erwinia herbicola* is sometimes referred to as *Enterobacter agglomerans*.

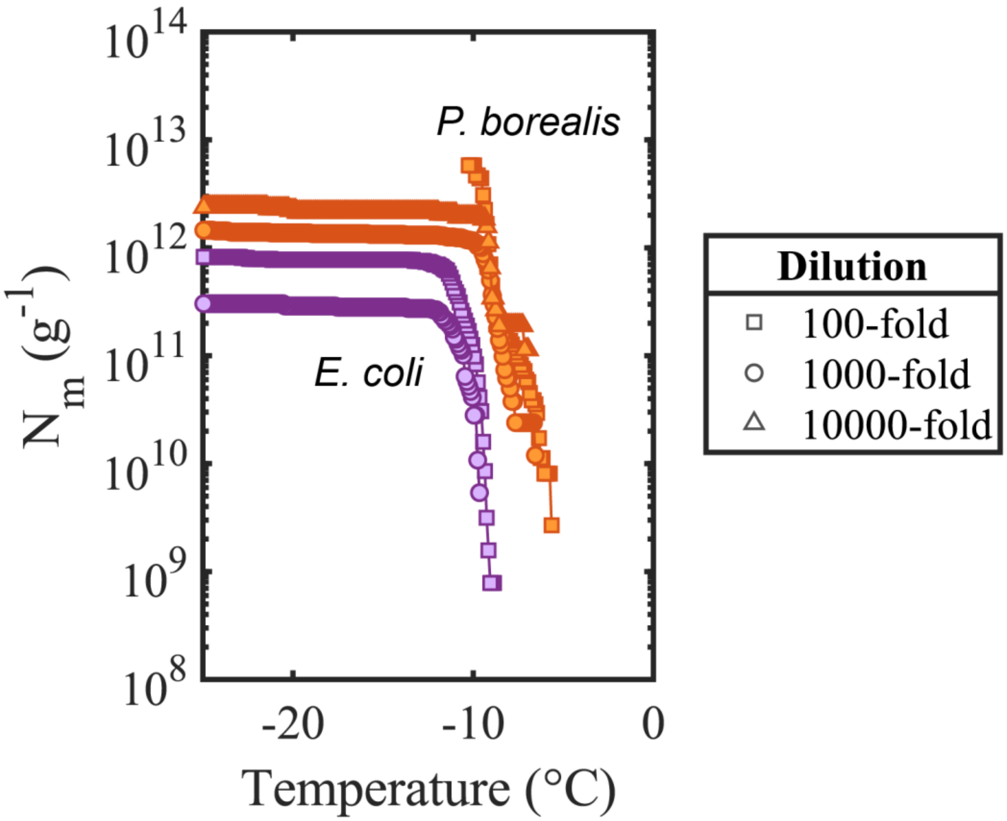

Supplementary Figure 2. Ice nucleation of diluted intact bacteria. Data were replotted from Figure 4 to show the cumulative number of ice nucleators per bacterial mass (Nm) as a function of temperature, where 1.0 g equates to about $10^{\wedge 12}$ bacteria. Data points for *P. borealis* are coloured orange with dilutions of 100-fold, 1000-fold and 10000-fold indicated by open squares, circles, and triangles, respectively. Data points for *E. coli* are coloured purple with dilutions of 100-fold and 1000-fold indicated by open squares and circles, respectively.

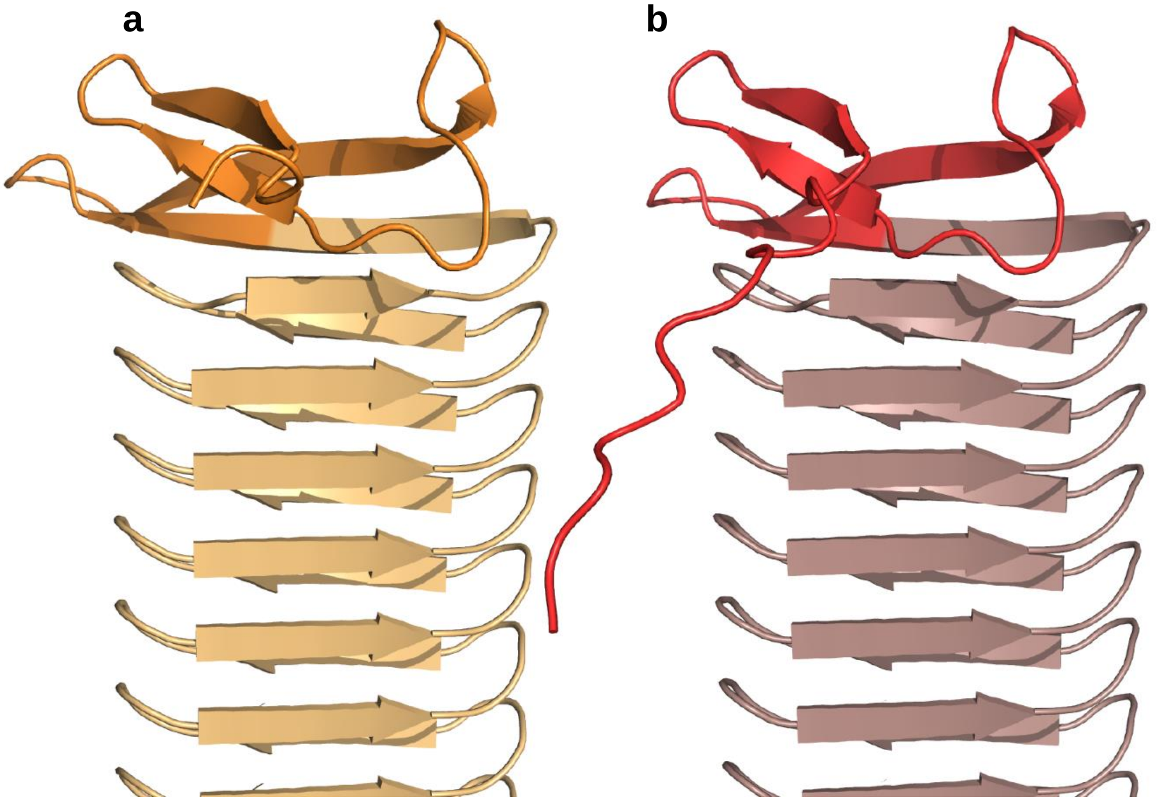

Supplementary Figure 3. AlphaFold predicted structures for the C-terminal regions of *Pb*INP and InaZ. a C-terminal end of *Pb*INP showing the cap domain in orange and the adjacent solenoid coils in yellow. **b** C-terminal end of InaZ (*Pseudomonas syringae*) showing the cap domain in red and the adjacent solenoid coils in brown.

Supplementary Table 1. DNA sequences involved in the production of *Pb*INP constructs discussed in Results.

Associated construct(s)	Type of DNA	Oligonucleotide sequence
19-repeat *Pb*INP	Converter to enable insertion of mutant DNA segment	Forward: 5' [PO_4^{2-}] – CGCTGACTGCAGGTTATGGCTCTACTCAAACTGCTCAAG AGGGGAGCT<br>Reverse: 5' [PO_4^{2-}] – CCCCTCTTGAGCAGTTTGAGTAGAGCCATAACCTGCAGT CAGCGAGCT
R9-mRuby2-*Pb*INP	Primers (F + R)	Forward: 5'– GATCGGTACCATGGTGTCGAAG – 3'<br>Reverse: 5' – CTAGGGTACCTTTGTAGAGCTCATCCA – 3'
15-to-18-repeat *Pb*INP	Synthesized gene fragment	5'– ACTAGTGGCT TCGCTAGCAG CCTGATTGCC GGTTACGGCA GCACCCAGAC GGCCGGCTAT GAGAGCACTC TCACGGCGGG TTACGGAAGT ACCCAGACGG CGGAACGTGA CAGCACATTA ACCGCCGGTT ATGGATCCAC GGGTACGGCC GGCCAGGATA GCAGCCTGAT CGCCGGTTAT GGGTCGACCC TGACCAGCGG TATTCGTAGC TTCCTGACGG CGGGTTACGG GTCGACCCTG ATTAGCGGCC TGCGTAGCGT GTTAACTGCG GGCTACGGAT CCAGCTTAAC TAGT – 3'

Supplementary Note 1. Calculation for estimation of the number of nucleators in a drop.

We assume that a drop contains 0, 1, or more nucleators, and, if there are no nucleators in a drop, that it will freeze at the homogenous temperature. We can use the Poisson distribution to estimate the number of nucleators in a drop[1].

The probability for N nucleators in a drop, where M is the mean of number of nucleators in a drop is: $P(N, M) = \frac{M^N e^{-M}}{N!}$

The probability to have zero nucleators in a drop is therefore: $P(0) = e^{-M}$

As we measure $P(0) = 0.6$ (in Fig. 3b, 10^{-3} dilution), we can calculate: $M = -ln(0.6) = 0.51$

Thus, the probability for one nucleator is: $P(1) = M * e^{-M} = 0.51 * 0.6 = 0.306$

The probability to have one or more nucleators is: $P(> 0) = 1 - P(0) = 0.4$

Thus, $P(1)/P(> 0) = 0.765$, or 76% of the drops that froze heterogeneously contain only one nucleator.

Supplementary References

1. Harbron EJa, Barbara PF. The Poisson distribution and single-molecule spectroscopy. An undergraduate analytical laboratory experiment. *Journal of Chemical Education* **79**, 211 (2002).
