# Supplementary material for: Water-organizing motif continuity is critical for potent ice nucleation protein activity
Source: Nat Commun. 2022 Aug 26;13:5019. doi: 10.1038/s41467-022-32469-9 (PMC9418140; doi:10.1038/s41467-022-32469-9)
Supplement: Supplementary file 3 — Reporting Summary [file 41467_2022_32469_MOESM3_ESM.pdf]

nature portfolio

Corresponding author(s): Dr. Peter L Davies

Last updated by author(s): Aug 10, 2022

Reporting Summary

Nature Portfolio wishes to improve the reproducibility of the work that we publish. This form provides structure for consistency and transparency in reporting. For further information on Nature Portfolio policies, see our Editorial Policies and the Editorial Policy Checklist.

Statistics

For all statistical analyses, confirm that the following items are present in the figure legend, table legend, main text, or Methods section.

n/a	Confirmed	
☐	☒	The exact sample size (*n*) for each experimental group/condition, given as a discrete number and unit of measurement
☐	☒	A statement on whether measurements were taken from distinct samples or whether the same sample was measured repeatedly
☒	☐	The statistical test(s) used AND whether they are one- or two-sided<br>*Only common tests should be described solely by name; describe more complex techniques in the Methods section.*
☒	☐	A description of all covariates tested
☒	☐	A description of any assumptions or corrections, such as tests of normality and adjustment for multiple comparisons
☒	☐	A full description of the statistical parameters including central tendency (e.g. means) or other basic estimates (e.g. regression coefficient) AND variation (e.g. standard deviation) or associated estimates of uncertainty (e.g. confidence intervals)
☒	☐	For null hypothesis testing, the test statistic (e.g. *F*, *t*, *r*) with confidence intervals, effect sizes, degrees of freedom and *P* value noted<br>*Give P values as exact values whenever suitable.*
☒	☐	For Bayesian analysis, information on the choice of priors and Markov chain Monte Carlo settings
☒	☐	For hierarchical and complex designs, identification of the appropriate level for tests and full reporting of outcomes
☒	☐	Estimates of effect sizes (e.g. Cohen's *d*, Pearson's *r*), indicating how they were calculated

Our web collection on statistics for biologists contains articles on many of the points above.

Software and code

Policy information about availability of computer code

Data collection	-Molecular Evolutionary Genetics Analysis (MEGA) software (version 11.0.10) was used to produce the consensus-matching residues plot in Fig. 5a<br>-SeaView Multiplatform GUI for molecular phylogeny (version 5.0.5) was used to produce the multiple sequence alignment for INPs shown in Supplementary Figure 1
Data analysis	-ColabFold (version 1.3.0), produced by AlphaFold creators, was used to analyze a raw protein sequence and output atomic coordinates

For manuscripts utilizing custom algorithms or software that are central to the research but not yet described in published literature, software must be made available to editors and reviewers. We strongly encourage code deposition in a community repository (e.g. GitHub). See the Nature Portfolio guidelines for submitting code & software for further information.

Data

Policy information about availability of data

All manuscripts must include a data availability statement. This statement should provide the following information, where applicable:

- Accession codes, unique identifiers, or web links for publicly available datasets
- A description of any restrictions on data availability
- For clinical datasets or third party data, please ensure that the statement adheres to our policy

The ice nucleation assay and AlphaFold model data generated in this study, alongside the synthesized PbINP gene sequence, have been deposited in the figshare

database [https://doi.org/10.6084/m9.figshare.20341440.v1].

Human research participants

Policy information about studies involving human research participants and Sex and Gender in Research.

Reporting on sex and gender	*Use the terms sex (biological attribute) and gender (shaped by social and cultural circumstances) carefully in order to avoid confusing both terms. Indicate if findings apply to only one sex or gender; describe whether sex and gender were considered in study design whether sex and/or gender was determined based on self-reporting or assigned and methods used. Provide in the source data disaggregated sex and gender data where this information has been collected, and consent has been obtained for sharing of individual-level data; provide overall numbers in this Reporting Summary. Please state if this information has not been collected. Report sex- and gender-based analyses where performed, justify reasons for lack of sex- and gender-based analysis.*
Population characteristics	*Describe the covariate-relevant population characteristics of the human research participants (e.g. age, genotypic information, past and current diagnosis and treatment categories). If you filled out the behavioural & social sciences study design questions and have nothing to add here, write "See above."*
Recruitment	*Describe how participants were recruited. Outline any potential self-selection bias or other biases that may be present and how these are likely to impact results.*
Ethics oversight	*Identify the organization(s) that approved the study protocol.*

Note that full information on the approval of the study protocol must also be provided in the manuscript.

Field-specific reporting

Please select the one below that is the best fit for your research. If you are not sure, read the appropriate sections before making your selection.

[x] Life sciences [ ] Behavioural & social sciences [ ] Ecological, evolutionary & environmental sciences

For a reference copy of the document with all sections, see nature.com/documents/nr-reporting-summary-flat.pdf

Life sciences study design

All studies must disclose on these points even when the disclosure is negative.

Sample size	During the calibration and validation of the WISDOM apparatus (Reicher, Segev, and Rudich, Atmos. Meas. Tech., 11, 233–248, 2018), it was established that measuring the freezing of ~50 droplets provided representative data for the T50 of ice nucleation in a sample. Each WISDOM experiment assayed the freezing of approximately 120 droplets, ranging from 80-100 µm in diameter. For samples examined in our study, WISDOM ice nucleation experiments were repeated at least twice, each time using a different microfluidic chip. This repetition verifies technical details such as device/chip integrity and sample suspension homogeneity. In addition, samples were also measured on BINARY in triplicate with 64 droplets per assay for each sample. This cross-referencing on two different apparatuses confirmed the temperature and range of sample ice nucleation activity.
Data exclusions	No data were excluded from the analyses.
Replication	The ice nucleation data were verified as described above, using several dozen sample droplets, duplicate/triplicate trials, and assays on WISDOM as well as BINARY. Sample freezing data were highly reproducible, both within and between apparatuses. These samples were sent in a blind manner and their identity was only revealed after the samples had been assayed. Each sample shipment also contained a blind ‘internal control’ construct that served as an additional measure of reproducibility.
Randomization	This is not relevant to our experiments. Each sample has its own genetic identity and was analyzed individually using consistent protocols, and therefore they did not require separation into experimental groups.
Blinding	Samples were assayed on the WISDOM and BINARY apparatuses without the operators knowing sample identity. After measurements were completed, the genetic identities of the samples were revealed.

Reporting for specific materials, systems and methods

We require information from authors about some types of materials, experimental systems and methods used in many studies. Here, indicate whether each material, system or method listed is relevant to your study. If you are not sure if a list item applies to your research, read the appropriate section before selecting a response.

Materials & experimental systems

n/a	Involved in the study
☒	☐ Antibodies
☒	☐ Eukaryotic cell lines
☒	☐ Palaeontology and archaeology
☒	☐ Animals and other organisms
☒	☐ Clinical data
☒	☐ Dual use research of concern

Methods

n/a	Involved in the study
☒	☐ ChIP-seq
☒	☐ Flow cytometry
☒	☐ MRI-based neuroimaging
